# Supplementary material for: Control of complex systems with generalized embedding and empirical dynamic modeling
Source: PLoS One. 2024 Aug 1;19(8):e0305408. doi: 10.1371/journal.pone.0305408 (PMC11293753; doi:10.1371/journal.pone.0305408)
Supplement: Attachment — Submitted filename: Reviewer_2_Rev1.pdf [file pone.0305408.s001.pdf]

Reviewer #2:

1) The novelty of the work can be expressed in detail in the Abstract, Highlights, and Conclusion. The main content should concern your research idea and its experimental effect verification.

> Please note we have highlighted the novel contribution in the Abstract, Introduction and Conclusion.

2) The expression of the abstract should be improved. A more detailed presentation of innovation should be conducted as well as the experimental verification.

> Thank you for this comment. One is always faced with the constraint of an Abstract word limit and the desire to communicate details of the innovation and experiments. Nonetheless, your comment is well received and we have added detail to the Abstract. Please note the PLoS One submission criteria include: "The Abstract should not exceed 300 words".

3) Recent related work survey is currently missing. More recent literature should be cited and analyzed, such as (a) Double internal loop higher-order recurrent neural network-based adaptive control of the nonlinear dynamical system, (b) Memory Recurrent Elman Neural Network-Based Identification of Time-Delayed Nonlinear Dynamical System, (c) Modeling and adaptive control of nonlinear dynamical systems using radial basis function network, (d) A novel feed-through Elman neural network for predicting the compressive and flexural strengths of eco-friendly jarosite mixed concrete: design, simulation and a comparative study, (e) Self-recurrent wavelet neural network–based identification and adaptive predictive control of nonlinear dynamical systems, (f) Externally Recurrent Neural Network based identification of dynamic systems using Lyapunov stability analysis and so on.

> Thank you for this guidance and comment. We added additional subsections in the Introduction with this, and other, state-of-the-art progress.

4) The expression logic can be improved for your proposed method so that the innovation can be clearly understood. More expression of the mathematical analysis should be conducted to show your ideas clearly. Also, please try to highlight your proposed method and focus on it.

> Thank you for this comment. We have clarified the text regarding the innovation and added a mathematical description of the projection method.

5) Grammar should be checked and improved for the entire content. Please try to make every sentence to be correct and easy to be understood.

> We have reviewed and rewritten the manuscript to improve the communication and simplicity of the sentences.

6) More experimental result comparisons with references should be conducted for advantage discussion.

> We added a model comparison to a neural network model and discussion of the advantages of the proposed method.

7) What about the stability of the proposed method? It is not discussed. You can refer to the following papers for your reference in the introduction and discuss the stability issue: (a)Lyapunov stability-based control and identification of nonlinear dynamical systems using adaptive dynamic programming, (b) Temporally local recurrent radial basis function network for modeling and adaptive control of nonlinear systems, (c) Diagonal recurrent neural network-based adaptive control of nonlinear dynamical systems using Lyapunov stability criterion, (d) A Lyapunov-stability-based context-layered recurrent pi-sigma neural network for the identification of nonlinear systems.

> Thank you for this comment and observation. Please note the method described is agnostic regarding the controller, which is mentioned in the manuscript. The innovation is use of generalized embedding and state space projection as the model predictive component of model predictive control. Regarding the controller used in the example, stability is verified with a Laplace domain analysis which has been added to the manuscript. It is also noted in the manuscript that every trial conducted out of hundreds the controller performed well and did not diverge.
