# Supplementary material for: Control of complex systems with generalized embedding and empirical dynamic modeling
Source: PLoS One. 2024 Aug 1;19(8):e0305408. doi: 10.1371/journal.pone.0305408 (PMC11293753; doi:10.1371/journal.pone.0305408)
Supplement: Attachment — Submitted filename: Reviewer_3_Rev2.pdf [file pone.0305408.s002.pdf]

Reviewer #3:

1. The article announces the treatment of the modeling and control problem for complex nonlinear dynamical systems. However, apart from a narrative description of the significance of the targeted research topic there are no specific findings and no contribution to the nonlinear control and to nonlinear estimation area. The modeling and system identification part is missing while the control part of the article is absent too. Considering the absence of specific results the article has to be substantially rewritten after taking into account major findings and established nonlinear control and nonlinear estimation methods in the subject area.

Response:

Regarding the perception “there are no specific findings and no contribution to the nonlinear control and to nonlinear estimation area” we assert that contributions to nonlinear control and nonlinear estimation may be found in:

Abstract : In many control applications this knowledge is expressed mathematically or through data-driven models, however, as complexity grows obtaining a satisfactory mathematical representation is increasingly difficult. Further, many data-driven approaches consist of abstract internal representations that may have no obvious connection to the underlying dynamics and control, or, require extensive model design and training. *Here, we remove these constraints by demonstrating model predictive control from generalized state space embedding of the process dynamics providing a data-driven, explainable method for control of nonlinear, complex systems.* Generalized embedding and model predictive control are demonstrated on nonlinear dynamics generated by an agent based model of 1200 interacting agents. The method is generally applicable to any type of controller and dynamic system representable in a state space

Lines 23-27 : Here we demonstrate the use of generalized state space embedding {Deyle2011} as a data--driven process model representation and leverage empirical dynamic modeling (EDM) {EDM-Wikipedia, Chang2017} as a nonlinear predictor applied to complex system control.

Lines 33-37: Advantages of generalized state space embedding and EDM are 1) The process model is data--driven and encapsulated in the dynamical state space, 2) Information flow from observations to predictions is deterministic and traceable, 3) Extensive parameterization or training are not required.

Line 138-142: Further, the direct connection between observation functions and the generalized embedding means there is a deterministic, traceable chain of information from observations to model predictions, a feature that is commonly absent from neural network models.

Lines 160-163: It can be noted these methods have non-trivial complexity as the error surface, adaptive control laws, and fault compensator are computed and updated at each time step, steps that are not required in the proposed generalized embedding approach.

Lines 189-198: We note several distinctions from methods discussed above:

1. The data defines the state space. There is no a-priori specification of candidate library functions or dictionary
2. Predictions are made directly in the state space
3. Direct interpretability is maintained since there is no external representation, such as a deep-learning model or functional abstractions
4. Recursive optimization is not needed
5. Methods to assess cross variable interactions in the dynamics are inherent in EDM facilitating direct inspection of multivariate dependencies of the dynamics and control.

Line 207-209: Here, we employ cross mapping with EDM sequential locally weighted global linear maps (s--map) {Sugihara-1994} to predict a feedback control variable.

Line 216-217: We capitalize this feature to infer dynamical dependencies of the state space and control variable.

Line 221-223: The innovation demonstrated here is application of generalized state space embedding and empirical dynamic modeling as a process model for model predictive control.

In reference to “there are no specific findings”, consider :

Section 4. Results.
Section 4.2 Model comparison.
Figure 5, 6, 7. Table 1. These figures and table provide explicit results demonstrating the nonlinear control and mechanistic explanation of intervariable interactions.

In reference to “The modeling and system identification part is missing while the control part of the article is absent too” please consider :

The modeling and system identification is detailed in sections 3.2, 3.3 and 3.4. As stated in the manuscript, the process dynamics are generated by an ABM, the process dynamics are modeled in a generalized embedding state-space, model predictive control is provided by empirical dynamic modeling.

2. Regarding the modeling part one would expect results on nonlinear least squares approaches or on nonlinear Kalman Filtering techniques. The modeling part is a prerequisite for the control part. In the case of model-based control precise estimation of the parameters of the dynamic model of the controlled system can be performed with the use of nonlinear Kalman Filtering or nonlinear least squares algorithms. In the case of mode-free control, identification of the dynamic model can be performed with the training of nonlinear regressors such as neural or neurofuzzy networks.

Thank you for this description of techniques employed for MPC. Please note we explicitly discuss these in:

Section 1.8 Kalman–Takens filter
Section 1.5 Artificial neural network

3. Regarding the nonlinear control of the targeted complex systems on would expect results on global linearization-based control methods or on approximate linearization-based control schemes Global linearization-based control uses transformations of the system's state variables and of the system's state-space model into equivalent linear forms where the control and estimation problem can be treated with solutions initially designed for linear systems. Approximate linearization-based control techniques considers Taylor series expansion of the system's dynamics around specific operating points through the computation of Jacobian matrices. The control and estimation problems are solved sequentially around the linearization points

Thank you for this encapsulation of linearization techniques applied to MPC. Please note we explicitly discuss state-of-the-art linearization techniques in :

Section 1.1 Chaos control
Section 1.3 Dynamic mode decomposition
Section 1.4 Sparse identification of nonlinear dynamics.
